# The Match between Molecular Subtypes, Histology and Microenvironment of Pancreatic Cancer and Its Relevance for Chemoresistance

**DOI:** 10.3390/cancers13020322

**Published:** 2021-01-17

**Authors:** Javier Martinez-Useros, Mario Martin-Galan, Jesus Garcia-Foncillas

**Affiliations:** Translational Oncology Division, OncoHealth Institute, Health Research Institute-Fundacion Jimenez Diaz University Hospital, Av. Reyes Catolicos 2, 28040 Madrid, Spain; mariomgtics@gmail.com

**Keywords:** molecular subtypes of pancreatic cancer, microenvironment, chemotherapy response, pancreatic stellate cells, regulatory T cells, tumor-associated macrophages, myeloid derived suppressor cells

## Abstract

**Simple Summary:**

Based on the four molecular subtypes of pancreatic cancer described by Bailey et al. 2016, in the present article we match the molecular, histology and microenvironment features of pancreatic cancer. This approach may help to understand the molecular basis of this kind of tumor, and how their microenvironment may affect treatment response. Moreover, we compile information about crucial factors that could serve as potential targets for drug design to achieve higher anti-tumor activity, and how histological evaluation of tumor microenvironment could provide first signs about treatment response.

**Abstract:**

In the last decade, several studies based on whole transcriptomic and genomic analyses of pancreatic tumors and their stroma have come to light to supplement histopathological stratification of pancreatic cancers with a molecular point-of-view. Three main molecular studies: Collisson et al. 2011, Moffitt et al. 2015 and Bailey et al. 2016 have found specific gene signatures, which identify different molecular subtypes of pancreatic cancer and provide a comprehensive stratification for both a personalized treatment or to identify potential druggable targets. However, the routine clinical management of pancreatic cancer does not consider a broad molecular analysis of each patient, due probably to the lack of target therapies for this tumor. Therefore, the current treatment decision is taken based on patients´ clinicopathological features and performance status. Histopathological evaluation of tumor samples could reveal many other attributes not only from tumor cells but also from their microenvironment specially about the presence of pancreatic stellate cells, regulatory T cells, tumor-associated macrophages, myeloid derived suppressor cells and extracellular matrix structure. In the present article, we revise the four molecular subtypes proposed by Bailey et al. and associate each subtype with other reported molecular subtypes. Moreover, we provide for each subtype a potential description of the tumor microenvironment that may influence treatment response according to the gene expression profile, the mutational landscape and their associated histology.

## 1. Introduction

Pancreatic cancer (PC) incidence has increased in developed countries and its trend for 2030 is to be higher reaching the second cause of cancer-related deaths [1]. When tumors are <2 cm in size, the 5-year survival rate is around 50% while for tumors <1 cm could average as much as 100% [2]. Regrettably, pancreatic cancer symptoms are often misdiagnosed and commonly treated ambulatory that leads to a late diagnosis with metastatic disease in distant organs; then, their 5-year survival rate decreased to 3% [3,4]. PC could be detected by elevated levels of CA19-9 serum marker [5]. However, CA19-9 is not PC specific and its levels are also high after biliary obstruction [6]. Recently, CA19-9 serum level in combination with IGF-1 and albumin have increased the sensitivity up to 93.6% and the specificity up to 95% to identify PC patients [7].

Risk factors of PC are cigarette smoking associated to 20–25% cases [8,9], chronic pancreatitis (4%) [10], diabetes (30%) [11], and some infectious agents like *Helicobacterpylori* (65%) presents an increased risk [12].

Surgical resection is considered the best treatment approach against PC. Histopathological characteristics of resected tumor like margins of resection (R), differentiation of tumor cells (G) or lymph-node status (N) could predict patient prognosis [13]. After resection, adjuvant treatment will depend on patient performance status but is usually based on gemcitabine [14], 5-fluorouracil and their combination with other cytotoxics [15]. The combination of gemcitabine with capecitabine exhibited better survival rates [16]. Other regimens based on FOLFIRINOX (folinic acid, 5-fluorouracil, irinotecan and oxaliplatin) or gemcitabine in combination with nano-albumin-bound paclitaxel (nab-paclitaxel) is administered to borderline resectable tumors as neoadjuvant treatment [17]. Another option for R1 patients, borderline resectable or locally advanced unresectable tumors is chemoradiotherapy [18,19]. However, PC exhibits chemoresistance due to a complex link between tumor cells and their microenvironment [20]. The tumor microenvironment TME is referred to the local environment where tumors developed [21]. The TME of PC is composed by tumor cells, extracellular matrix and stromal cells (pancreatic stellate cells (PSCs), regulatory T cells (Tregs), tumor-associated macrophages (TAMs), and myeloid derived suppressor cells (MDSCs). All these TME components foster high levels of hypoxia, which confer several metabolic advantages to tumor cells. Some studies have shown how PC microenvironment regulates proliferation, invasion and metastasis, chemoresistance and immune evasion [22,23].

Microenvironment of PC has immunosuppressive characteristics that lead to escape from the immune system, which enhances tumor progression. Cell populations from TME could induce the deposition of extracellular molecules such as matrix metalloproteinases, extracellular matrix molecules, growth factors and transforming growth factor β (TGF-β) to maintain the enabling microenvironment [24]. PSCs are able to produce a collagenous stroma and its role is crucial in both normal pancreas and tumor development. PSCs cooperate with cancer cells to build a perfect niche of tumor development with invasive abilities [25]. While in a normal physiological state, PSCs are quiescent and express nestin, vimentin, GFAP and desmin; activated PSCs become myofibroblast-like cells with overexpression of collagen type I and III, fibronectin and laminin, and present high proliferation and migration abilities [26]. Furthermore, activated PSCs allow pancreatic cancer-related fibrosis [27]. Interestingly, PSCs with an activated phenotype present expression of α-SMA [26], while CD10 positive PSCs have the ability to promote an invasive phenotype in PC [28]. There are subgroups of PSCs that interact with pancreatic *β-cells* called islet stellate cells. These islets are able to induce *β-cell* apoptosis, inhibit their proliferation, and diminish *β-cell* function [29,30]. Indeed, activated PSCs could impair pancreatic islet endocrine function. Since PSCs are glucose intolerant, this cell population is involved in development of type 2 diabetes, which directly promotes invasive phenotype because high levels of glucose are related to epithelial-to-mesenchymal transition [29,31]. PSCs promote also epithelial-to-mesenchymal transition of PC tumor cells [26].

Another TME cell population is Tregs that are a CD4+T cells subpopulation generated from naïve T cells with expression of FOXP3 and CD25.They are present in TME and they are able to suppress autoimmunity in physiological conditions, but also inhibit anti-tumor immune response. This fact could be explained because Tregs can impact tumor-associated CD11c+ dendritic cells to inhibit the activation of CD8+ T cells [32]. Recently, it has been described how Tregs induce differentiation of cancer-associated fibroblasts that promote tumor development [33]. Shevchenko et al. reported that gemcitabine at low doses reduce Tregs population in PC and provide a modest increase in survival [34]. Another study described how neutrophil-lymphocyte ratio and a high proportion of Treg cells in pancreatic tumors are potential pathological biomarkers for poor outcome [35]. On the other hand, TAMs imply a subtype of immune cells that are commonly expressed in several solid tumors, and closely related to cancer-derived inflammation. Specially M2-polarized TAMs (CD163+ cells) are those cells with direct implication in cancer-derived inflammation; in addition, M2-polarized TAMs enable epithelial-to-mesenchymal transition through activation of TLR4/IL-10 signaling and inhibition of E-cadherin [36]. Additionally, TAMs promote angiogenesis, dedifferentiation and stem-cell phenotype in PC [37]. TAMs show several tumor-prone characteristics based on the release of some immunosuppressive and angiogenic cytokines [38]. In PC, TAMs present a high expression of BMP-4, BMP-7, TGF-β1 and TGF-β2 [39]. The infiltration of M2-polarized TAMs in PC stroma seems to be preferentially located in the body and tail of the pancreas, and similarly with other TME cell populations, M2-polarized TAM confers shorter overall survival to patients [40]. It has been reported how metformin could reduce desmoplasia of PC, reversion of epithelial-to-mesenchymal transition, and tumor-related inflammation via modulation of the AMPK/STAT3 that decreases levels of IL-1β and hampers infiltration of M2-polarized TAMs [41]. Indeed, metformin in combination with simvastatin and digoxin is being evaluated in a phase IB trial to target crucial factors for PC development like PDX1 or BIRC5 (NCT03889795) [42]. In PC, M2-polarized TAM population rises significantly after gemcitabine administration, and it has been reported how aspirin could improve the effects of gemcitabine and decrease not only M2-polarized TAMs but also MDSCs populations [43]. MDSCs are a very heterogeneous immature myeloid cell population derived from the myeloid lineage that act as a multipotent progenitor cells. They are involved in development of obesity and several pathologies like autoimmune disease, chronic inflammation and tumorigenesis [44], and they are characterized by the expression of CD11b and Gr-1 surface markers [45]. Sangaletti et al. revealed how downregulation of SPARC decreased immunosuppression and reverted invasive phenotype triggered by the presence of MDSCs [46]. Immunosuppression by MDSCs is also driven by downregulation of JAK3, MHC class II and STAT5 that inhibit activation of T-cells or induce their apoptosis [44]. Another study revealed that the pro-inflammatory microenvironment that promotes MDSCs proliferation and recruitment is produced by a cytokine cascade, which includes the release of IL-1β, IL-6, IL-13, IL-17, TNF-α, TGF-β and VEGF [47]. Similarly, other factors such as ARG-1, COX-2, Cybb, Cytochrome b-245, iNOS2 and PAUF are related to MDSCs activation [48]. MDSCs also impair chemo- and radiotherapy response. In fact, high levels of proinflammatory cytokines associated with MDSCs have been found in previously treated versus untreated patients or healthy samples [49]. Since radiotherapy induces the release of lactate by tumor cells that lead to activation of MDSCs, a proposed treatment strategy would imply target lactate to increase radiotherapy response [50]. Another mechanism described recently against MDSCs is by omega 3 administration due to its anti-inflammatory properties. Indeed, the combination of omega 3 with gemcitabine drastically stabilizes Tregs population and decreases MDSCs in advanced PC [51]. Other treatment approaches, like anti-CXCR2, have been described to counteract MDSCs [52], or an anti-ENO1 that limits the invasion of MDSCs and causes the subsequent disruption of TME interactions with tumor cells, which may improve survival or PC patients [53].

Other studies have proposed the administration of triterpenoid to hamper the activation of MDSCs and their immunosuppressive action in PC, which lead to an increased immune surveillance and immune response [54,55]. Therefore, diminish MDSCs population is a potential target for the treatment of PC and increase its chemoradiosensitivity.

Molecularly, PC is characterized by inactivating mutations in *TP53*, *RB*, *CDKN1A*, *CDKN2A*, or those associated to heritable PC such as *BRCA1* and *BRCA2* [56,57]. The effect of tumor suppressor genes could be blinded by overexpression of other oncogenes like *MYC*, *CCNE1* or *RAF* [58]. Broad genomic analysis has revealed different subtypes of PC. Bailey et al., identified four molecular subtypes of PC associated with specific histopathological characteristics: Squamous, progenitor, immunogenic and aberrantly differentiated endocrine/exocrine (ADEX) [59]. The squamous molecular subtype is associated to squamous differentiation, TP63N activation, inflammation, hypoxia, metabolic reprogramming, dense extracellular matrix, TGF-β and WNT signaling pathway, increased proliferation, activation of MYC, autophagy and enhanced RNA processing. Progenitor and immunogenic subtypes share several characteristics like activation of FOXA2 and FOXA3 networks, xenobiotic metabolism, fatty acid oxidation and mucins metabolism. However, immunogenic subtype also involves the presence of several immune pathways like B cell, CD4 and CD8 T cell signaling, TLR signaling, and other factors associated with antigen presentation. ADEX is characterized by upregulation of NR5A2, RBPJL transcriptional regulation pathway, exocrine differentiation, development of pancreatic *β-cell*, epithelial cells differentiation and presents mutations in *KRAS*. Moffitt et al., proposed other molecular subtypes based on analyses of patient prognostic and gene expression profile of tumors (classical or basal-like tumors) and stroma (normal or activated stroma) compared with healthy samples [60]. Collisson et al., also proposed three molecular subtypes by a transcriptional profile analysis: Classical, which overlaps with Moffitt´s classical subtype and exhibit high expression of molecules necessary for cell adhesion and epithelial morphology; quasi-mesenchymal subtype that shows high expression levels of genes associated to mesenchymal morphology; and exocrine-like subtype with overexpression of genes required for digestive enzymes release [61]. These diverse molecular subtypes exhibit several similarities with each other and highlight several opportunities for novel therapeutic strategies and targeted drug design.

TME of PC should be different according to different molecular subtypes and their response to chemotherapy should be different too. In the present article, we review the four molecular subtypes of pancreatic cancer proposed by Bailey et al., and we used them as backbone to supplement their features with other PC molecular subtypes proposed by Moffitt et al., and Collisson et al. Furthermore, we match each molecular subtype to a specific tumor histology and microenvironment, and how each complex network may influence treatment response.

## 2. Squamous Subtype

**Figure 1:**
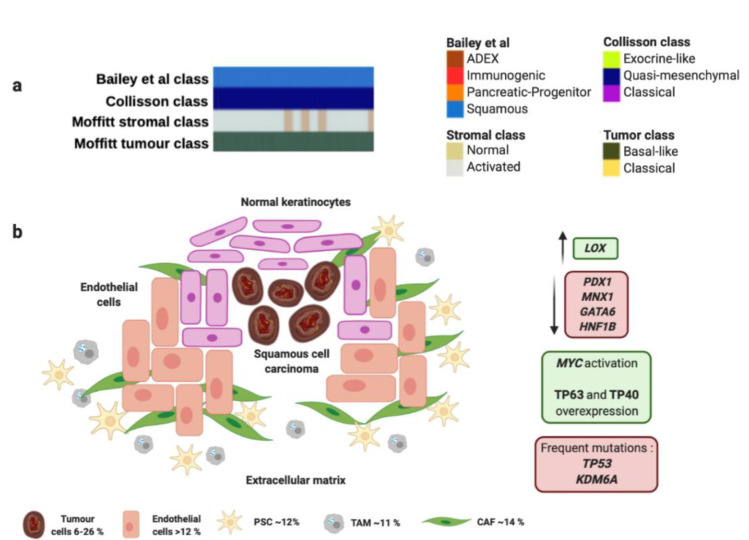
Squamous molecular profile and microenvironment. (**a**) Squamous subtype is associated with specific histological characteristics such as squamous and adenosquamous carcinomas. (**b**) Squamous subtype proposed by Bailey et al., matches perfectly with "quasi-mesenchymal" tumor subtype proposed by Collisson et al., and “basal-like” tumors and “activated” stroma subtypes proposed by Moffitt et al. Green boxes contain upregulated significant factors and activated pathways associated to squamous molecular subtype. Red boxes contain downregulated significant factors (up) and most common mutations found in squamous molecular subtype (down). Percentage of each subcellular population has been obtained from Peng et al., [86]. Abbreviations: TAM: Tumor-associated macrophage; CAF: Cancer-associated fibroblast; PSC: Pancreatic stellate cell.

Squamous and adenosquamous tumors represent 1–4% of all PCs, are often generated in the head of the pancreas [62], and present the worst survival ratios even detected at early stages [63]. These tumors frequently carry *TP53* mutations and present 3p loss compared to adenocarcinomas (Figure 1 and Table 1). Losses in 3p21.1–11.1 imply downregulation in several anti-oncogenic genes like *ROBO1*, *ROBO2, WNT5A, FHIT*. The squamous histology also presents upregulation of the WNT/β-Catenin signaling pathway and downregulation of some chromatin modification factors [64]. These tumors are characterized by several cell layers with irregular borders, protruding intercellular junctions, high eosinophilic cytoplasm and keratin deposition, with denotes a rich and dense extracellular matrix (Figure 1 and Table 1) [65].

The squamous molecular subtype proposed by Bailey et al., presents MYC activation and overexpression of proteins TP63 and CD7 *(TP40)* as well as its target factors [59]. This molecular subtype was associated with presence of *TP53* and *KDM6A* mutations [59,66].

Interestingly, overexpression of TP63 in combination with *TP53* mutation is able to induce epithelial-to-mesenchymal transition to promote aggressive phenotype [67]. Squamous tumors also express integrins α6β1, α6β4 and EGF. Concerning epigenetic modifications, this subtype exhibits an hypermethylator phenotype that downregulates protein expression of PDX1, MNX1, GATA6 or HNF1B (Figure 1 and Table 1). However, it presents upregulation of LOX, which is associated with metastasis development [68]. Squamous molecular subtype exhibit an hypoxic, inflamed and autophagic microenvironment that correlates with squamous histology [59]. Moreover, Moffitt et al. found a squamous subtype characterized by overexpression associated with several components of the extracellular matrix like laminins and keratins [60]. This matrix composition allows an oncogenic microenvironment that fosters proliferation of PC tumor cells. This dense extracellular matrix generates a physical barrier for drug delivery that leads to a chemoresistant phenotype [69]. This rich extracellular matrix contributes to generation of hypoxic conditions and expression of hypoxia-inducible factor 1 (HIF1) [70], and overexpression of MUC1, VEGF and PDGF that enhance the endothelial tube formation, proliferation and migration ability [71]. The squamous molecular subtype also presents activation of TGF-β signaling pathway [59], and overexpression of EGFR and S100A2 [72]. These findings support the presence and important role of PSCs in the development of squamous tumor microenvironment since cytokines TGF-β, PDGF, Angiotensin II could bind to the PSCs receptors and activate downstream signaling pathways such as ERK, c-Jun, P38, MAPK or JAK-STAT to promote the activation and proliferation of PSCs. Moreover, activated PSCs can produce and secrete to stroma a variety of growth factors through paracrine mechanism, to activate EGFR, PI3K-AKT, and mTOR signaling pathways, and then promote proliferation and inhibition of apoptosis of PC cells [73,74]. One study has reported that squamous PC cells are able to recruit neutrophils that convert PSCs into tumor-associated fibroblasts (TAFs) that overexpress inflammatory cytokines like IL1A and CXCL1 [75]. TAFs also contribute to generate a rich tumor prone extracellular matrix by secretion of fibroblast activating protein (FAP) that participate in angiogenesis and affect the activity of IFN-γ and TNF-α [76].

On the other hand, the squamous molecular subtype proposed by Bailey et al. linked with “basal-like” and “activated” stroma signatures proposed by Moffitt et al. (Figure 1 and Table 1). The “basal-like” and “activated” stroma signatures also exhibited the worst survivals compared to “classical” and “normal” stroma subgroups, which goes in accordance with both squamous molecular subtype of Bailey et al., and the squamous histology of PC [60]. “Basal-like” subtype presents *KRAS^G12D^* mutation, while “activated” stroma subtype expresses SPARC, WNT family members WNT2 and WNT5A, gelatinase B (MMP9), stromelysin 3 (MMP11), and FAP that contribute to tumor development [60]. Furthermore, “activated” stroma subgroup is characterized by other factors like the chemokine ligands CCL13 and CCL18, and integrin ITGAM, which are associated with the presence of macrophages [60]. This fact suggests the important role of macrophages in the tumor microenvironment to promote angiogenesis and chemoresistance [77]. In this sense, it has been described how TAMs enable chemoresistance to gemcitabine and nab-paclitaxel by expression of insulin growth factor (IGF) that activates IGF-1 receptors [78]. It has been reported that PSCs also promote gemcitabine resistance mediated by secretion of deoxycytidine that modulate nucleoside transporters [79]. Moreover, PSCs reduce the sensitivity of PC cells to radiotherapy by regulation of epithelial-to-mesenchymal transition and increasing cancer stem cell markers [80]. Furthermore, these authors provide a rationale for the use of an anti-TGF-β neutralizing antibody to inhibit epithelial-to-mesenchymal transition and cancer stem cells to sensitize cells to radiation and reduce tumors [80]. Other cell population like tumor associated fibroblasts can secrete proinflammatory factors like IL-6, which activates STAT3, and secrete CXCL12 that inhibit T-cells infiltration in tumors [81]. In addition, CXCL12 in combination with anti-PD-L1 immunotherapy has shown a synergic anti-tumor effect and is considered a potential target for those PCs with high FAP expression [82].

Unfortunately, Bailey et al., support that squamous molecular subtype exhibits the highest expression of PD-L1, PD-L2 and IDO1 [59], thus, they sustain that these tumors are not good candidates for immunotherapy administration [83,84].

However, the combination of IDO1 inhibitor and anti-PD-1/PD-L1 treatment could achieve promising results and become a potential strategy to induce intratumoral T-cell infiltration [85]. In the light of the foregoing, the microenvironment of the squamous molecular subtype presents a complex interaction between different cell populations and extracellular components to confer chemoresistance of PC.

## 3. Progenitor and Immunogenic Subtypes

**Figure 2:**
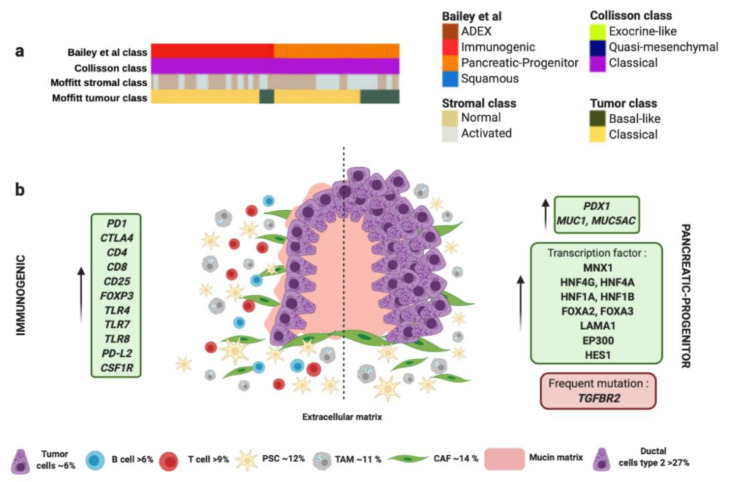
Immunogenic and progenitor molecular profiles and their microenvironment. (**a**) Both subtypes proposed by Bailey et al., match perfectly with “classical” tumor subtype proposed by Collisson et al., and “classical” tumor subtype proposed by Moffitt et al. (**b**) Both subtypes share the same histology derived from premalignant lesions like mucinous non-cystic adenocarcinomas and intraductal papillary mucinous neoplasms (IPMN) characterized by high mucin production. Dotted line separates schematic representation of two subtypes. Green boxes contain upregulated significant factors associated to each subtype. Red box contains the most characteristic gene mutation in progenitor molecular subtype. Pink color represents mucin production. Percentage of each subcellular population has been obtained from Peng et al., [86]. Abbreviations: TAM: tumor-associated macrophage; CAF: cancer-associated fibroblast; PSC: pancreatic stellate cell.4. Microenvironment of ADEX Subtype.

The progenitor molecular subtype proposed by Bailey et al. presents activation on transcriptional networks that exhibit high expression of the proteins HES1, HNF1A, HNF1B, HNF4A, HNF4G, FOXA2, FOXA3, LAMA1, MNX1 and EP300 (Figure 2 and Table 1) [59]. These transcription factors are considered crucial for determination and development of the pancreatic endoderm. While the squamous subtype presented downregulation of PDX1, which is also critical for pancreatic development, the progenitor subtype overexpress PDX1 (Figure 2 and Table 1) [88]. Moreover, progenitor molecular subtype expresses multiple genes necessary for the metabolism of mucins, fatty acids, steroid hormones, and also involves drug metabolism. Indeed, progenitor molecular subtype overexpresses MUC5AC and MUC1 (Figure 2 and Table 1) [59]. Interestingly, both progenitor and immunogenic molecular subtypes share the same histology derived from premalignant lesions like mucinous non-cystic adenocarcinomas and intraductal papillary mucinous neoplasms (IPMN), characterized by a high mucin production (Figure 2 and Table 1) [106], different epithelial-to-mesenchymal transition markers [107], and immunohistochemical staining of CDX2 [89,90].

The immunogenic molecular subtype also presents many other similarities in the gene expression profile with the progenitor subtype; however, immunogenic subtype exhibit a significantly higher immune infiltrate [59]. Gene expression analysis identified upregulation in those genes associated with nine different populations of immune cells [108], being the most significant those involved with B and T cells infiltration (CD4, CD8, CD25, FOXP3) (Figure 2 and Table 1). Furthermore, immunogenic subtype presents activation of CTLA4 and PD1, which suggests that this molecular subtype could be feasible treated with immunecheckpoints inhibitors [59]. In contrast, the pancreatic progenitor subtype presents the lowest levels of CTLA4, PD-L1, PD-L2, and IDO1 [59]. The immunogenic subtype also has enrichment in genes for Toll-like receptors that play a key role in the innate immune system. These genes include TLR4, TLR7, TLR8, PD-L2 and CSF1R (Figure 2 and Table 1) [59]. These receptors usually are expressed on sentinel cells such as macrophages and dendritic cells, which reveal the strong link between the immunogenic molecular subtype with the presence of immune cell population in the TME (Figure 2 and Table 1). In this sense, TAMs present in PC, especially those M2-type macrophages can promote neovasculature formation to feed tumor cells and induce chemoresistance, proliferation, invasion and metastasis of PC tumor cells [77]. TAMs could be recruited to neoplasic foci by cytokines and vascular endothelial growth factors (VEGF). Once there, TAMs promote proliferation of PC cells through the release of growth factors like Il10 or TGF-β [97]. In addition, the presence of TAMs in the TME is associated with chemoresistance [78].

On the other hand, the immunogenic molecular subtype of PC present inflammation, caused by abundant Tregs infiltration and high expression of cytotoxic T lymphocyte-associated protein 4 (CTLA-4). These factors promote tumor immunosuppression through the inhibition of the killing ability of effector cells [95]. The presence of CD8+ T cells in immunogenic subtype can decrease the capability of anti-tumor immune cells due to the ability of CD8+T cells to express FAS-ligand and secrete perforin and granzymes with cytolytic effect [96]. Despite the immunosuppressive skills of immunogenic subtype, patients with this kind of tumor present the best outcome out of the four molecular subtypes [59].

Interestingly, both progenitor and immunogenic subtypes correlate with “classical” tumor subtype proposed by Moffitt et al., which also share the expression of extracellular mucin in more than 10% (Figure 2 and Table 1) [60]. Similarly to immunogenic molecular subtype, “classical” tumors present better outcome compared to those “basal-like” tumors [60]. Remarkably, progenitor and immunogenic molecular subtypes by Bailey et al. and “classical” tumor subtype by Moffitt et al., correlate with the same gene expression signature of “classical” tumor subtype proposed by Collisson et al. (Figure 2 and Table 1) [109]. This fact supports the presence of the G12V variant of *KRAS* mutation that is associated with the “classical” subtype [60]. “Classical” molecular subtype also presents enrichment in genes associated with GATA6 overexpression responsible for promoting epithelial cell differentiation [60]. However, other authors propose the loss of GATA6 expression in tumors confers a shorter survival and they have a poor response to adjuvant treatment based on 5-fluorouracil (5-FU)/leucovorin [91]. In this sense, “classical” molecular subtype tumors also exhibit poor response to platinum based therapy [60]. Therefore, the adjuvant treatment used for the management of immunogenic/progenitor/classical molecular subtype tumors would be more aggressive to overcome the chemoresistance. For this, immunotherapy has arisen as one of the most effective anti-tumor treatments. PC overexpress important factors for targeted therapies, such as MUC-1, CEA, PSC, VEGF, MSLN and mutation *KRAS* [110]. However, the most important targets for immunotherapy currently are PD-1, PD-L1 and CTLA-4. In animal models, anti-PD-1 or anti-PD-L1 treatment boosted infiltration of CD8+ T cells in the tumor and enhanced efficacy of immunocheckpoint inhibitors. 

Regretfully, immunotherapy against PC has presented no benefit in clinical trials so far [111,112]. Currently, the failure of anti-PD-1/anti-PD-L1 based therapy is mainly attributed to the lack of T-cell infiltration in the TME of PC. Therefore, the immunogenic molecular subtype that presents elevated genes for B- and T-cell infiltration is a potential responder for immunotherapy. Other type of immunotherapy based on CAR-T cells has obtained promising results. Some clinical trials have focused on modified CAR-T cells able to target MUC1 or Mesothelin (MSLN) [113]. Although MUC1 is overexpressed in approximately 85% of PC, those tumors present overexpression of IDO1, COX1/2, and Gal-9 that conferred resistance to these anti-MUC1 CAR-T cells [114]. However, the use of inhibitors of IDO1, COX1/2, and Gal-9 overcame this resistance in combination with anti-MUC1 CAR-T cells [114]. In this concern, a phase I/II trial has been designed with resectable PC patients´ T cells modified to allow identification and kill of MUC1 positive tumor cells (NCT02587689). CAR-T cells could be engineered to target other antigens such as Mesothelin, and they have achieved encouraging results in solid malignancies [115], which has established the bases for a subsequent clinical trial with anti-MSLN CAR-T cells (NCT04203459). Although CAR-T cell therapy has achieved favorable results, it is crucial to evaluate the potential side effects on T-cell exhaustion.

**Figure 3:**
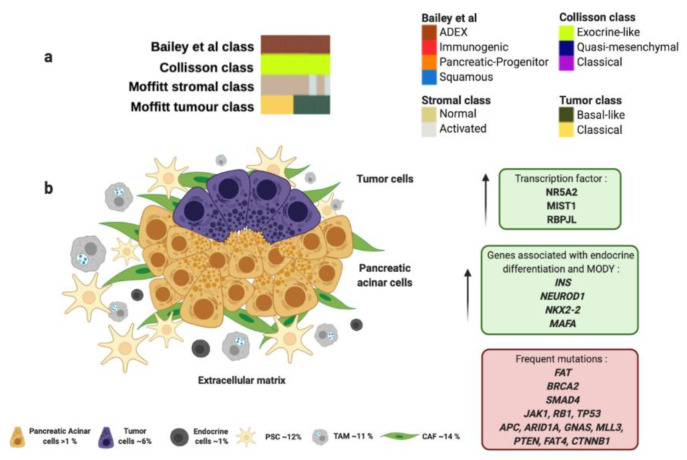
ADEX molecular profile and microenvironment. (**a**) ADEX subtype proposed by Bailey et al., matches perfectly with “exocrine-like” subtype proposed by Collisson et al., and most of “normal stroma” subtype cases analyzed by Moffitt et al. (**b**) ADEX molecular subtype is associated with the rare acinar histology. Green boxes contain upregulated transcription factors and other genes involved in ADEX molecular subtype. Red box contains genes commonly mutated in ADEX. Percentage of each subcellular population has been obtained from Peng et al., [86]. Abbreviations: TAM: tumor-associated macrophage; CAF: cancer-associated fibroblast; PSC: pancreatic stellate cell; MODY: maturity onset diabetes of the young.

The ADEX molecular subtype by Bailey et al., is defined by the orchestrated expression of factors necessary for the exocrine and endocrine pancreatic cell-fate determination. ADEX presents upregulation in transcription factors involved in healing and regeneration after pancreatitis events such as RBPJL, NR5A2, MIST1 and their associated downstream cascade factors (Figure 3 and Table 1) [100,101]. This molecular subtype exhibits an expression profile associated with extremely rare acinar histopathology (Figure 3 and Table 1) [59,63]. Macroscopically, this histology shows large tumors, frequently presented in encapsulated form and well circumscribed. It also presents cystic evolution, necrosis, and upper digestive hemorrhage [116]. In clinical practice, diagnosis of acinar carcinomas is performed by immunohistochemical evaluation of pancreatic enzymes like trypsin and chymotrypsin that are produced in the rough endoplasmic reticulum of acinar cells. It also presents high specificity for BCL-10 immunostaining [65]. Furthermore, somatic mutational index of acinar subtype is higher than adenocarcinoma being the most frequent mutations in the following genes: *FAT1, FAT3, and FAT4* (57%), *BRCA2* (42%), *SMAD4* (26%), *JAK1* (17%), *RB1* (13%), *TP53* (13%), *CTNNB1* (11%), *APC* (9%), *ARID1A* (9%), *GNAS* (9%), *MLL3* (9%), *PTEN* (9%), *RNF43* (4%) and *MEN1* (4%); however, *KRAS* mutations are not often observed in acinar subtype (Figure 3 and Table 1) [98,99]. In this sense, Bailey et al. found in ADEX molecular subtype high expression levels of *INS*, *NEUROD1*, *NKX2-2* and *MAFA,* which are associated with endocrine differentiation and sudden occurrence of diabetes type MODY (Figure 3 and Table 1).

Importantly, most PC-derived cell lines present enrichment in genes found in ADEX molecular subtype like *AMY2B*, *CEL*, *INS*, *PRSS1* and *PRSS3* [59].

ADEX molecular subtype proposed by Bailey et al., matches perfectly with “exocrine-like” subtype proposed by Collisson et al., which shows relatively high expression of tumor cell-derived digestive enzymes like ELA3A and CFTR, which controls Cl^−^ and Na^+^ ions, and water flux (Figure 3 and Table 1) [61]. In addition, ADEX molecular subtype correlates with “normal stroma” subtype proposed by Moffitt et al., which exhibit longer survival rates (Figure 3 and Table 1) [60]. “Normal stroma” is also distinguished by high expression levels of PSCs markers: Smooth muscle actin (ACTA2), Desmin (DES) and Vimentin (VIM) [60,102]. This fact highlights the importance of the PSCs population in the ADEX microenvironment. PSCs role go in accordance with ADEX origin, since they present expression markers of stem and progenitor cells needed to maintain differentiation and regeneration of pancreas [117]. Indeed, PSCs are necessary to trigger desmoplastic reaction by the synthesize of large amounts of extracellular matrix proteins, such as collagens, and amplify endostatin production of cancer cells that contributes to an hypoxic milieu [103,118]. Moverover, PSCs produce VEGF to increase endothelial cell growth in the peritumoral stroma [103]. All the above supports the role of PSCs in induction of fibrosis [119], that impairs drug delivery, stimulates epithelial-to-mesenchymal transition, and increases genetic instability leading to a more chemoresistant tumor [104]. Furthermore, PSCs play a crucial role in islet cell dysfunction since PSCs decrease insulin expression and an increased apoptosis of pancreatic *β-cells* that connects to initiation of diabetes [105]. One study supported that PC cells cultured with extracellular matrix proteins produced by PSCs were able to develop resistance to 5-FU, cisplatin and doxorubicin [120]. As above mentioned, the presence of PSCs in ADEX molecular subtype could imply resistance to gemcitabine [79] and a reduced sensitivity to radiotherapy [80].

## 4. Conclusions and Future Perspectives

The match between molecular features at genomic, epigenomic and expression levels combined with the biological behavior of PC is bringing new therapeutic strategies for the clinical management of these patients. In addition, the study of the TME could explain chemoresistance of PC previously attributed only to tumor cells. The TME and especially the role of its immune cell population in tumor initiation, development and immune evasion is changing the paradigm of PC diagnosis and treatment approach with the identification of new molecular subtypes (Table 2).

Although treatment strategies based on immunocheckpoint inhibitors have shown disappointing results, new studies are ongoing with novel drug design to improve patient response, and to avoid tumor immune evasion. In this concern, a randomized clinical trial (NCT02030860) aimed to evaluate paricalcitol that target vitamin D metabolism in the TME in combination with gemcitabine plus nab-paclitaxel in the neoadjuvant scenario for resectable PC patients. Another phase I/II clinical trial is designed to evaluate the safety and tolerability of the microenvironment modifier drug, L-DOS47, in combination with doxorubicin. The L-DOS47 is reported to neutralize the acid extracellular matrix that protects the tumor (NCT04203641) [121]. Recently, other treatments like GVAX or CRS-207 have appeared with promising results against PC [122]. GVAX is a vaccine based on granulocyte-macrophage colony-stimulating factor that inhibits Tregs and induces T cells; while CRS-207 is a vaccine based on live-attenuated *Listeria monocytogenes*–expressing mesothelin that has been described to induce innate and adaptive immune response against tumor cells and has achieved longer survivals of PC patients (NCT03161379; NCT02451982) [122]. Other drugs are based on nucleic acid, this is the case of olaptesed pegol a L-RNA Spiegelmer that exhibits a high affinity against CXCL12, a factor previously described responsible for TAMs recruitment (NCT03168139) [123]. These new treatment approaches highlight the importance of targeting not only tumor cells but also TME. Although translational research and drug design are working synergistically to improve outcome of PC patients, clinical research must not ignore that patients might be stratified not only based on performance status of patients and/or the molecular characteristics of tumors, but also according to their TME features to obtain better treatment responses and longer survivals.

## Figures and Tables

**Table 1:** Molecular features of each pancreatic cancer subtype according to their main subcellular population.

Bailey et al. Molecular Subtype	Cellular Subpopulations	Molecular Profile of Cellular Subpopulation	Expression of Cytokines, Chemokines and Receptors	Clinical Significance
Squamous	Tumor cells	*TP53* and *KDM6A* mutations [59,66]; *KRAS-G12D-* mutation [60]; overexpression of LOX [68]; upregulation of TP63, TP40 and IDO1 [59]; activation of WNT/β-Catenin pathway [64], *MYC* activation [59]; overexpression of HIF1 [70], MUC1 and PDGF [71], ROBO1, ROBO2, WNT5A, FHIT [64]; downregulation of PDX1, MNX1, GATA6 and HNF1B [59];	Laminins and keratins [60]; PD-L1 y PD-L2 [59]; CEACAM6, MUC1, EPCAM, CEACAM5, MMP7, CEACAM1, IL18, VEGFA, IL32 [86]	Immunotherapy resistance and aggressive phenotype [83,84]
	Stellate cell	Activation of ERK, c-Jun, P38, MAPK, JAK-STAT, PI3K-AKT, mTOR and cancer stem cell markers [73,74]; overexpression of RGS5, ACTA2, PDGFRB, SOD3, NOTCH3, COL18A1, COL4A1, NDUFA4L2, COL4A2, SEPT4, COL14A1 and SPARC [86]	Integrins α6β1, α6β4 and EGF [59]; TGF-β [59]; Angiotensin II and deoxycytidine [79]	Gemcitabine and radiotherapy resistance [79,80]
	Endothelial cells	Overexpression of COL15A1, RAMP2, CDH5, SPARCL1, NOTCH4, SMAD9 and JAM2 [86]	VEGF [71]	Angiogenesis [87]
	Macrophages	Overexpression of AIF1, APOC1, APOE, C1QB, C1QA, C1QC, NCF2 and CCL2 [86]	IGF, CCL13, CCL18 and integrin ITGAM [60,78]; HLA-DRA, HLA-DPA1, HLA-DPB1, CD14, CD53, CD68, CD74, FTH1, FTL, TGFBI, IL8 and IL13RA1 [86]	Enable chemoresistance to gemcitabine and nab-paclitaxel [78]
	Fibroblasts	SFRP2, LUM, COL1A1, COL1A2, DCN, COL3A1, MMP11, COL6A3, COL10A1, COL5A2, SPARC, COL6A2, COL5A1, COL6A1, COL8A1, COL12A1, MMP14, FGF7, FAP [86]	EGFR and S100A2 [72]; IL1A and CXCL1 [75]; IFN-γ, TNF-α, IL-6, CXCL12 and FAP [76,81,82]	Inhibit T-cells infiltration activity [85]
Progenitor	Tumor cells	*KRAS-G12V-* mutation [60]; *TGFBR2* mutation [59]; overexpression of MUC5AC and MUC1 [59]; upregulation of HES1, HNF1A, HNF1B, HNF4A, HNF4G, FOXA2, FOXA3, LAMA1, MNX1 and EP300 [59]; overexpression of PDX1 [88], GATA6 [60] and CDX2 [89,90]; downregulation of IDO1 [59], LOX and S100A [59]	High mucin production [60]; low levels of EGFR, CTLA4, PD-L1 and PD-L2 [59]	Chemoresistance, immune evasion and upregulation of cell survival pathways [91,92]; poor response to platinum-based therapy [60]
	Ductal cells type 2	Overexpression of SOX9 [86]	CEACAM1, CEACAM5, CEACAM6, EPCAM, IL18, IL32, KRT19, MMP7, MUC1, MUC5AC, VEGFA [86,93]	Poor clinical prognosis [86]
	Tumor cells	*KRAS-G12V-* mutation [60]; Overexpression of CDX2 [89,90] and GATA6 [60]		*KRAS-G12V-* highlights the presence of Tregs infiltration [94]; GATA6 expression inhibits cell dissemination [91] and confers poor response to platinum based therapy [60]
Immunogenic	B cells	Upregulation of LIMD2, IRF8, BANK1 and RAC2 [86]	CD19, CD20, CD37, CD52, CD53, CD74, CD79A, CD79B, CXCR4, IL16 and VPREB3 [86]	High B cells infiltration [86]
	T cells	Upregulation of FOXP3 [59]; Upregulation of RAC2 [86]	CD4, CD8, CD25, CTLA4 and PD1 [59]; CCL5, CD2, CD3E, CD3D, CD3G, CD7, CD45, CD52, CD69, CXCR4, IL2RG, IL7R, IL32, KLRB1, LTB [86]	Promote tumor immunosuppression [95]; CD8 cells secrete perforin and granzymes with cytolytic effect [96]
	Macrophages	Upregulation of AIF1, APOC1, APOE, C1QB, C1QA, C1QC, FTH1, NCF2 [86]	IL10 and TGF-β [97]; CSF1R, TLR4, TLR7, TLR8, PD-L2 [59]; CD14, CD53, CD68, CD74, CCL2, HLA-DRA, HLA-DPA1, HLA-DPB1, IL8, IL13RA1, TGFBI, and VEGFR [86]	Chemoresistance [78]
	Tumor cells	Presence of mutations in: *FAT1, FAT3*, and *FAT4* (57%), *BRCA2* (42%), *SMAD4* (26%), *JAK1* (17%), *RB1* (13%), *TP53* (13%), *CTNNB1* (11%), *APC* (9%), *ARID1A* (9%), *GNAS* (9%), *MLL3* (9%), *PTEN* (9%), *RNF43* (4%) and *MEN1* (4%); however, *KRAS* mutations are scarce [98,99]; upregulation of AMY2B, CEL, INS, PRSS1, PRSS3 [59]; and RBPJL, NR5A2, MIST1 [100,101]		Better clinical outcome [60]
ADEX	Acinar cells	Expression of BCL-10 [65]	IL32, PRSS1, REG1A, REG1B, REG3A, REG3G, [86]; ELA3A and CFTR [61]	Exhibit high exocrine features [65]
	Stellate cell	Overexpression of ACTA2, DES and VIM [60,102]; COL18A1, COL4A1, COL4A2, COL14A1, NDUFA4L2, NOTCH3, RGS5, SEPT4, SOD3 and SPARC [86]	VEGF [103] and PDGFRB [86]	Impairs drug delivery, stimulates epithelial-to-mesenchymal transition, increases genetic instability and chemoresistance [104]; increased apoptosis of pancreatic *β-cells* [105]
	Endocrine cells	Overexpression of INS, NEUROD1, NKX2-2, MAFA [59], and ABCC8 [86]	CHGA, CHGB, IAPP, PCSK1N and TTR [86]	Endocrine/exocrine differentiation and association with initiation of diabetes [59]

**Table 2:** On-going clinical trials that include different subtypes of pancreatic cancer.

Clinical Trial	Study Type	Treatment	Patients	Aim
NCT03977233	Phase II	Neoadjuvant FOLFIRINOX (oxaliplatin, leucovorin, irinotecan, 5-FU)	Untreated patients with resectable, borderline resectable and unresectable locally advanced pancreatic ductal adenocarcinoma	Assess the impact of tumor and stromal molecular subtypes on the efficacy of neoadjuvant FOLFIRINOX
NCT02869802	Observational Prospective	First-line systemic chemotherapy with either FOLFIRINOX or cisplatin plus gemcitabine based regimens	Tumor samples, baseline and serial blood and urine samples from metastatic pancreatic ductal adenocarcinoma	Patients will undergo fresh tumor biopsy at study enrollment for comprehensive molecular characterization
NCT04246710	Observational Prospective	Not specified	Resectable, borderline resectable, locally advanced, metastatic and recurrent pancreatic cancer	Molecular characterization and identification of markers for therapeutic stratification by endoscopic ultrasound tissue core biopsies
NCT04683315	Phase II	Patients with “classical” subtype will receive FOLFIRINOX; and patients with “basal” subtype will receive gemcitabine/nab-paclitaxel.	Patients with “classical” or “basal” subtype pancreatic cancer	Molecular subtyping of pretreated endoscopic ultrasound fine needle aspiration samples to determine pancreatic cancer subtype
NCT03820921	Observational Prospective	PD-1 inhibitor (pembrolizumab)	Unresectable or metastatic, microsatellite instability-high (MSI-H) or mismatch repair-deficient (dMMR) pancreatic cancer that have progressed following prior treatment and have no satisfactory alternative treatment options.	Assess of tumor PD-L1/dMMR expression in patients with pancreatic cancer using endoscopic ultrasound fine needle aspiration biopsy samples, and the prospective correlation of MMR status and PD-L1 expression with overall survival and progression-free survival of PDAC patients.
NCT04436679	Observational Retrospective	None	Excreto-pancreatic adenocarcinoma of the pancreas, ductal adenocarcinoma or “classical” adenocarcinoma	Analyze the microscopic characteristics of the stroma, tumor budding and mucin expression using a comparative approach of long-survivor/short-survival patients.
NCT03840460	Observational Prospective	Not specified	Early and advanced pancreatic adenocarcinoma, precursor lesions or pancreatic neuroendocrine tumors.	Study the molecular profile of pancreatic lesions and their microenvironment at various stages to predict treatment response, treatment toxicity and prognostic. Additionally, investigate the particular micro-organisms colonizing individual patients and association with patient’s outcome
